# Analysis of the Interaction between Globular Head Modules of Human C1q and Its Candidate Receptor gC1qR

**DOI:** 10.3389/fimmu.2016.00567

**Published:** 2016-12-13

**Authors:** Lina Pednekar, Ansar A. Pathan, Basudev Paudyal, Anthony G. Tsolaki, Anuvinder Kaur, Suhair M. Abozaid, Lubna Kouser, Haseeb A. Khan, Ellinor I. Peerschke, Mohamed H. Shamji, Gudrun Stenbeck, Berhane Ghebrehiwet, Uday Kishore

**Affiliations:** ^1^Biosciences, College of Health and Life Sciences, Brunel University London, London, UK; ^2^Department of Biochemistry, College of Science, King Saud University, Riyadh, Saudi Arabia; ^3^Department of Laboratory Medicine, Memorial Sloan-Kettering, Cancer Center, New York, NY, USA; ^4^Allergy and Clinical Immunology, National Heart and Lung Institute, Imperial College London, London, UK; ^5^Department of Medicine, State University of New York, Stony Brook, NY, USA

**Keywords:** C1q, globular head, gC1qR, protein–protein interaction, cell proliferation

## Abstract

The heterotrimeric globular head (gC1q) domain of human C1q is made up of the C-terminal ends of the three individual chains, ghA, ghB, and ghC. A candidate receptor for the gC1q domain is a multi-functional pattern recognition protein, gC1qR. Since understanding of gC1qR and gC1q interaction could provide an insight into the pleiotropic functions of gC1qR, this study was undertaken to identify the gC1qR-binding site on the gC1q domain, using the recombinant ghA, ghB, and ghC modules and their substitution mutants. Our results show that ghA, ghB, and ghC modules can interact with gC1qR independently, thus reinforcing the notion of modularity within the gC1q domain of human C1q. Mutational analysis revealed that while Arg162 in the ghA module is central to interaction between gC1qR and C1q, a single amino acid substitution (arginine to glutamate) in residue 114 of the ghB module resulted in enhanced binding. Expression of gC1qR and C1q in adherent monocytes with or without pro-inflammatory stimuli was also analyzed by qPCR; it showed an autocrine/paracrine basis of C1q and gC1qR interaction. Microscopic studies revealed that C1q and gC1qR are colocalized on PBMCs. Cell proliferation assays indicated that ghA, ghB, and ghC modules were able to attenuate phytohemagglutinin-stimulated proliferation of PBMCs. Addition of gC1qR had an additive effect on the anti-proliferative effect of globular head modules. In summary, our results identify residues involved in C1q-gC1qR interaction and explain, to a certain level, their involvement on the immune cell surface, which is relevant for C1q-induced functions including inflammation, infection, and immunity.

## Introduction

C1q is the first subcomponent of the classical pathway of the complement system that links innate and adaptive immunity by virtue of recognizing IgG and IgM in the immune complexes (1). Structurally, human C1q (460 kDa) is made up of 18 polypeptides, i.e., 6A, 6B, and 6C chains. Each chain has a short N-terminal region, a collagen-like region (CLR), and a C-terminal globular head (gC1q) domain (2). A combination of interchain disulfide bond formation and triple-helical CLR gives rise to an ABC–CBA structural subunit. Three of these subunits associate to yield the hexameric C1q molecule. The gC1q domain is a hetrotrimeric structure composed of C-terminal halves of A, B, and C chains. C1q is able to bind to an array of self, non-self, and altered-self ligands (3–7) *via* its gC1q domain (8). This ligand-binding versatility of C1q is offered by the modular organization of the individual globular head (gh) modules, ghA, ghB, and ghC, which are considered structurally and functionally independent (9–12).

A candidate receptor that binds to the gC1q domain of human C1q, called gC1qR (33 kDa), is a highly acidic, multi-ligand binding, and multi-functional protein. In addition to its role in the complement system, gC1qR is also involved in blood clotting *via* interaction with thrombin and vitronectin (13). Furthermore, as a high affinity receptor for high molecular weight kininogen and FXII, gC1qR present on the endothelial cells is able to serve as a major platform for the activation of the kinin/kallikrein, leading to the generation of the vasoactive peptide, bradykinin (14, 15).

Although the gC1q–gC1qR interaction has been described previously (16), the complementary binding sites and the precise nature of interaction remain to be fully established. The major gC1q-binding site on gC1qR has been shown to be located on residues 76–93 based on peptides studies (17). The availability of the recombinant individual gh modules, ghA, ghB, and ghC, which represent globular region of A, B, and C chains, respectively, without collagen region of C1q (18) has given us the opportunity to examine the gC1q–gC1qR interaction more closely. With respect to the structure/function relationship within the gC1q domain (19, 20), it is now known that ghA, ghB, and ghC are functionally independent modules. The modular organization of the gC1q domain offers C1q, the versatility required for binding to a range of self and non-self ligands. This is evident in the case of the HIV-1 gp41 peptide 601–613, which preferentially binds to ghA (20), and the β-amyloid peptide specifically interacting with ghB (20).

The crystal structure of gC1qR has revealed three monomers held together to form a trimer (21). Each monomer consists of seven anti-parallel β strands filled by an N-terminal and two C-terminal α helices. gC1qR has a distinct charge distribution, with the “solution face” of its “donut” shaped structure that is highly negatively charged and exposed to the plasma, while the reverse side or “membrane face” is neutral or basic (17). The C1q binding site, residues 76–93, is exposed only on the highly charged solution face (17). Since the C1q binding site on gC1qR has been identified, we sought to identify the complementary residues on the gC1q domain that are involved in the gC1q–gC1qR interaction. Previous studies have highlighted Arg^B114^ and Arg^B129^ of the B chain to be central in the C1q–IgG interaction (22). It has also been shown that C1q binding to gC1qR on platelets (23) and endothelial cells (24) induces complement activation independent of IgG. Furthermore, although gC1qR has been shown to bind to the gC1q domain of C1q, its physiological relevance still remains to be established.

Here, we have examined the interaction of recombinant forms of ghA, ghB, and ghC modules with gC1qR. We also used single residue substitution mutants for ghA, ghB, and ghC (19, 20, 22) that allowed us to identify residues on the gC1q domain that participate in the C1q–gC1qR interaction. A number of substitution mutants: ghA-R162A, ghA-R162E, ghB-R114A, ghB-R114Q, ghB-R163E, ghB-R163A, ghB-H117D, ghB-R129A, ghB-R129E, ghB-T175L, ghC-R156E, ghC-L170E, and ghC-H101A were tested for their interaction with gC1qR. The functional characterization of the point mutants identified an important role of Arg^162^ of ghA and Arg^114^ of ghB in the structure–function relationship involving C1q and gC1qR.

It is known that at sites of inflammation, adherent monocytes start to overexpress C1q. Thus, we performed a series of qPCR experiments to assess whether gC1qR expression was concomitant with C1q in adherent monocytes. gC1qR was upregulated, together with C1q on adherent monocytes, suggesting that both the ligand and the receptor are required under inflammatory conditions. The previously reported C1q-mediated anti-proliferative effect on T cells (25) could be reproduced qualitatively by the individual recombinant gh modules, which inhibited phytohemagglutinin (PHA)-stimulated proliferation of PBMCs. This anti-proliferative effect of gh modules was further enhanced by the addition of gC1qR, suggesting that gC1qR, in conjunction with C1q, can play an important role in modifying cellular immune responses.

## Experimental Procedures

### Purification of Human C1q

C1q was purified from freshly thawed plasma, as published earlier (26). Briefly, plasma was made 5mM EDTA, pH 7.5, and centrifuged at 12,000 × *g* to remove aggregated lipids. The plasma was then incubated with non-immune IgG coupled to CNBr-activated Sepharose (GE Healthcare, UK) for 1 h at 4°C. The plasma was filtered through a sintered glass funnel, and C1q bound to IgG–Sepharose was then washed extensively with 10 mM HEPES, 140 mM NaCl, 0.5 mM EDTA, and pH 7.0. C1q was eluted with *N*-cyclohexyl-3-aminopropanesulfonic acid (CAPS) buffer (100 mM CAPS, 1 M NaCl, 0.5 mM EDTA, pH 11). The eluted C1q was then passed through a HiTrap Protein G column (GE Healthcare) to remove IgG contaminants and dialyzed against the washing buffer.

### Expression and Purification of Wild-type ghA, ghB, ghC, and Substitution Mutants

The recombinant gh modules ghA, ghB, ghC, and their respective substitution mutants, were expressed in *Escherichia coli* BL21 fused to maltose-binding protein (MBP) in their monomeric forms (18). Bacterial cells were grown in 200 ml Luria–Bertani (LB) medium containing ampicillin (100 μg/ml) at 37°C on a shaker. Once grown to an OD_600_ of 0.6, the bacterial cells were induced with 0.4mM isopropyl β-d-thiogalactoside (IPTG) for 3 h and pelleted *via* centrifugation (4500 rpm for 15 min). The cell pellet was suspended in 25 ml of lysis buffer [20 mM Tris–HCl pH 8.0, 0.5 M NaCl, 1 mM EDTA, 0.2% v/v Tween 20, 5% glycerol, 0.1 mM phenylmethylsulfonyl fluoride (PMSF), and 100 μg/ml lysozyme] and incubated at 4°C for 1 h on a rotatory shaker. The cells were then sonicated (SoniPrep 150) at 60 Hz for 30 s with 2 min interval for 10 cycles. After centrifugation (13,000 rpm for 15 min), the supernatant was collected and diluted fivefold in buffer I (20 mM Tris–HCl, pH 8.0, 100 mM NaCl, 0.2% Tween 20, 1 mM EDTA, and 5% v/v glycerol) and passed through an amylose resin 15 ml bed column (New England Biolabs). The column was previously washed with three bed volumes of buffer I followed by buffer II (buffer I without Tween 20). The protein was then eluted in 1 ml fractions with 10 mM maltose in buffer II.

### Cloning, Expression, and Purification of Human gC1qR

Recombinant mature gC1qR (residues 74–282) (27) was expressed in *E. coli* BL21 (λDE3) (Life Technologies). Bacterial cells were grown in 250 ml of LB medium at 37°C until an OD_600_ of 0.6 was reached and protein expression was induced with 0.5 mM IPTG. Following another 3 h incubation on a shaker, bacterial culture was spun down (4500 rpm, 15 min). The cell pellet was treated with lysis buffer (20 mM Tris pH 8.0, 0.5 M NaCl, 1 mM EDTA, 0.2% v/v Tween, 5% v/v glycerol, and 100 μg/ml lysozyme) and incubated for 1 h at 4°C with mild shaking. The resulting cell lysate was sonicated, as described above for gh modules. The sonicate was spun down at 13,000 rpm for 15 min, and the collected supernatant was dialyzed for 2 h against 20 mM Tris–HCl, pH 7.5. The dialyzed protein was subjected to an ion exchange chromatography using a Q-Sepharose column (Sigma). gC1qR was step-eluted at 0.45 M NaCl. Although we did not determine the oligomeric state of the recombinant gC1qR, it is likely to be a trimeric structure, based on the crystallization studies (21).

The purified fractions were passed through Pierce™ High Capacity Endotoxin Removal Resin (Thermo Fisher) to remove lipopolysaccharides (LPSs). Endotoxin levels in the protein preparations were determined using the QCL-1000 Limulus amebocyte lysate system (BioWhittaker Inc., USA). The assay was linear over a range of 0.1–1.0 EU/ml (10 EU = 1 ng of endotoxin) and the amount of endotoxin present in the preparations was estimated to be <4 pg/μg of the recombinant protein.

### ELISA

Direct binding ELISA was performed to examine the interaction of C1q and gh modules with gC1qR. Microtiter wells (Maxisorp, Nunc) were coated with 1 μg of gC1qR (in 100 μl) in carbonate/bicarbonate buffer, pH 9.6, and left overnight at 4°C. Unbound proteins were removed and the wells were blocked with 2% w/v BSA in PBS for 2 h at 37°C. The plate was then washed three times with PBS + 0.05% Tween 20, and then different concentrations (2.5, 1.25, 0.625, and 0.312 μg/well) of ghA, ghB, or ghC modules (MBP as a control protein) were diluted in calcium buffer (50 mM Tris–HCl pH 8.0, 100 mM NaCl, and 5 mM CaCl_2_) and added to the wells. The plate was kept first at 37°C for 1 h and then at 4°C for another hour. Following further washes, the bound protein was detected by anti-MBP monoclonal antibody (Sigma) (1:5000 dilution in PBS) and probed with rabbit anti-mouse IgG Horseradish peroxidase (HRP; 1:5000; Promega; #W402b) in PBS. Color was developed using *o*-phenylenediamine dihydrochloride (OPD) substrate (Thermo-Fisher Scientific) and the plate was read at 450 nm using iMark Microplate Absorbance reader (Bio-Rad).

Microtiter wells were coated with different concentrations of human C1q (5, 2.5, 1.25, and 0.625 μg/well in 100 μl) in carbonate/bicarbonate buffer, pH 9.6, and left overnight at 4°C. Unbound proteins were removed and the wells were blocked with 2% w/v BSA in PBS for 2 h at 37°C. The plate was then washed three times with PBS + 0.05% Tween 20, and then the wells were incubated with 2.5 μg of gC1qR in calcium buffer (50 mM Tris–HCl, pH 8.0, 100 mM NaCl, and 5 mM CaCl_2_). The plate was kept first at 37°C for 1 h and then at 4°C for another hour. Following further washes, bound gC1qR was detected by anti-gC1qR polyclonal antibody (IgG fraction; 1:5000 dilution), followed by Protein A-HRP (1:5000 dilution) conjugate. MBP was used as a negative control protein.

### Western Blotting

The immunoreactivity of the recombinant gC1qR (10 μg/lane) was assessed by western blotting. Following a 12% v/v SDS-PAGE gel, the protein was electrophoretically transferred onto PDVF membrane, followed by blocking for 1 h at room temperature with 5% non-fat milk in PBS. Recombinant human gC1qR was probed with rabbit anti-human gC1qR polyclonal antibodies (IgG fraction; 500 μg/ml concentration; 1:1000 dilution) and incubated at 37°C for 1 h. The membrane was washed three times in 0.02% PBS–Tween 20, 30 min each and probed with Protein A-HRP conjugate (Sigma, 1:1000 dilution in PBS) for 1 h at room temperature. Color was developed using 3,3′-diaminobenzidine (DAB).

Far-western blot was carried out to test the interaction of ghA, ghB, ghC, and key substitution mutants with gC1qR. Ten micrograms of each protein was run on a SDS-PAGE gel, followed by transferring and blocking as described above. Fifteen micrograms per milliliter of either the gh’s modules or gC1qR was added in 10 ml calcium buffer (20 mM Tris–HCl, pH 7.5, 5 mM CaCl_2_, and 100 mM NaCl) and incubated overnight at 37°C. Membranes were washed and probed with either polyclonal anti-gC1qR or rabbit anti-MBP (Life Technologies, 1:1000) polyclonal antibodies which were diluted in 2% w/v non-fat milk powder in PBS and incubated for 2 h at 37°C. The blots were developed as described above.

### Fluorescence Microscopy

The binding of recombinant ghA, ghB, and ghC modules to gC1qR was examined microscopically using monocyte-derived human macrophages. Human PBMCs were separated from human blood from healthy volunteers (with ethical approval by the Institutional committee of Brunel University London) by Ficoll-paque (GE Healthcare) density gradient method. The separated PBMCs were suspended in complete medium (RPMI 1640, 2mM l-glutamine, 100 μg/ml Penicillin/Streptomycin, and 10% FCS). The 1 × 10^6^ cells were seeded on 13 mm diameter cover slips and incubated for 14 days at 37°C in 5% CO_2_ incubator.

PBMCs were treated with 10 μg of individual gh modules for 1 h at 37°C in serum-free medium. MBP (10 μg) was used as a negative control protein. Cells were then washed three times with PBS, fixed using 4% paraformaldehyde (PFA) for 10 min, and then rinsed with PBS three times. The coverslips were permeabilized using a buffer containing 20mM HEPES–NaOH pH 7.4, 300mM sucrose, 50mM NaCl, 3mM MgCl_2_, 0.5% Triton X-100, and 10% sodium azide for 5 min on ice, and then blocked with 5% FCS in PBS (wash buffer) for 30 min. The slides were then incubated with mouse anti-MBP (1:500 dilution in wash buffer) and rabbit anti-gC1qR antibodies (1:100 dilution in wash buffer) for 30 min. The slides were washed three times in wash buffer, 10 min each, and subsequently incubated with secondary antibodies: Alexa Fluor 647 conjugated donkey-anti-mouse antibody (Abcam; Cat: ab150111) 1:500 dilution in wash buffer and Alexa Fluor 488 conjugated goat anti-rabbit antibody (Abcam; Cat: ab150077) 1:500 dilution in wash buffer for 30 min. To stain the nucleus, Hoechst 33342 (Invitrogen, Cat: H3570 at 1:10,000 dilution) was used. The slides were then washed three times in the wash buffer 10 min each, mounted using Citifluor anti-fade (Citifluor, UK) and observed under a Leica DM4000 Fluorescent microscope using Leica Application Suite Software. In the merged images, the Alexa Fluor 647 color was set to red.

### Quantitative RT-PCR

Whole blood (50 ml) was taken from healthy volunteers and 2 units/ml of heparin sodium (product details – PL 29931/015) (Wockhardt), was added to prevent blood clotting. Blood was then diluted with an equal volume of RPMI 1640. To isolate monocytes, blood in RPMI 1640 was separated on a Ficol column (Ficol-Plaque Plus, GE healthcare) by centrifugation at 2000 rpm for 16 min at room temperature. The top layer was removed and PBMCs interphase layer was carefully removed. An equal volume of RPMI 1640 was then added and the cells were pelleted by centrifugation at 1500 rpm for 10 min at room temperature. Cells were then re-suspended in 50 ml of RPMI 1640 and the cell concentration was determined using a hemocytometer (total yield 7 × 10^7^ cells).

Then, 5 × 10^6^ cells were added to each tissue culture well in a 24-well plate in 5 ml of RPMI 1640 containing 10% FCS, 100 μg/ml penicillin-streptomycin, and 2 mM l-glutamine, and incubated at 37°C with 5% CO_2_ v/v atmosphere and left to adhere. Cells were then harvested at the following time points of incubation for adherence: 2 h, 24 h, 48 h, 72 h, 5 days, and 7 days. A similar experiment was also set up with the addition of 20 ng/μl of LPS (*Salmonella typhimurium*, Sigma-Aldrich). Adherent cells, with or without LPS, from each time point were harvested by removing the media from the plate and incubating cells in RPMI 1640 containing 0.025% trypsin/0.01% EDTA for 5 min at 37°C. Cells were removed using a cell scraper and an equal volume of RPMI 1640 containing 10% FCS was added to the harvested cells. Cells were pelleted by centrifugation at 1500 rpm for 10 min at room temperature and stored at −80°C until RNA extraction was carried out.

Total RNA was extracted using the GenElute Mammalian Total RNA Purification Kit (Sigma-Aldrich). Samples were then treated with DNase I (Sigma-Aldrich) to remove any contaminating DNA followed by heating at 70°C for 10 min to inactivate both DNase I and RNase, and then chilled on ice. The amount of total RNA was measured by determining the absorbance at 260 nm using the NanoDrop 2000/2000c (Thermo-Fisher Scientific) and the purity of the RNA was assessed using the ratio of absorbance at 260 and 280 nm. cDNA was synthesized using High Capacity RNA to cDNA Kit (Applied Biosystems) using 2 μg of total RNA.

Primer sequences were designed using the nucleotide Basic Local Alignment Search Tool and Primer (BLAST, http://blast.ncbi.nlm.nih.gov/Blast.cgi). The following primers were used: for 18S rRNA gene (endogenous control): forward (5′-ATGGCCGTTCTTAGTTGGTG-3′), reverse (5′-CGCTGAGCCAGTCAGTGTAG-3′); for C1q C chain gene: forward (5′-CAAAGGGCAGAAGGGAGAAC-3′), reverse (5′-ATCTGATCAGGCTGTTGGGT-3′); and for gC1qR gene: forward (5′-AACAACAGCATCCCACCAAC-3′), reverse (5′-AGATGTCACTCTCAGCCTCG-3′).

PCR was performed on all samples in order to assess the quality of cDNA. The qPCR reactions, performed for measuring the expression level of C1q and gC1qR, consisted of 5 μl Power SYBR Green MasterMix (Applied Biosystems), 75nM of forward and reverse primer, 500 ng template cDNA in a 10 μl final volume, using a 7900HT Fast Real-Time PCR System (Applied Biosystems). The initial steps were 2 min incubation at 50°C, followed by 10 min incubation at 95°C. The template was then amplified for 40 cycles under these conditions: 15 s at 95°C and 1 min at 60°C. Samples were normalized using the expression of human 18S rRNA. Data were analyzed using the Relative Quantification (RQ) Manager Version 1.2.1 (Applied Biosystems). Cycle threshold (Ct) values for each target gene were calculated and the relative expression was calculated using the RQ value *via* the formula: RQ = 2^−∆∆Ct^ for each target gene, and comparing relative expression with that of the 18S rRNA constitutive gene product. Assays were conducted twice in triplicate. Statistical analysis was performed using GraphPad Prism version 6.0 (GraphPad Software). An unpaired two-side *t*-test was used to compare the means of the expressed targets of the time points analyzed, using the 2 h time point as the calibrator. *p* Values were computed, and graphs compiled and analyzed.

### Cell Proliferation Assay

PBMCs were re-suspended in serum-free medium containing RPMI 1640, Penicillin/Streptomycin and Sodium Pyruvate, and stimulated with PHA (Sigma; 11249738001) at a concentration of 1 μg/ml and 1 × 10^5^ cells (100 μl) were aliquoted per well in a 96-well tissue culture plate. Next, the cells were treated with 20 μg/ml of ghA, ghB, ghC, or gC1qR in their respective wells. Different concentrations of gC1qR (1.25, 2.5, 5, and 10 μg) were also coincubated with 20 μg each of gh modules. At the 72 h time point, ^3^H-methy thymidine (MP Biomedicals, USA) was added and the plate was pulsed for 16 h. Cells were subsequently harvested using a semi-automated cell harvester and the amount of ^3^H-thymidine incorporated into DNA was measured using a liquid scintillation counter. Each experiment was conducted in triplicates.

## Results

### Expression and Purification of Recombinant Human gC1qR in *E. coli* under Bacteriophage T7 Promoter

**Figure 1:**
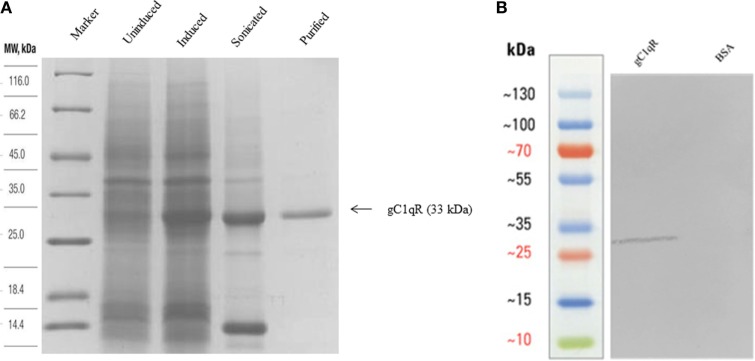
**(A)** Expression and purification of recombinant gC1qR. Twelve percent (v/v) SDS-PAGE under reducing conditions. *E. coli* BL21 (λDE3) strain, transformed with plasmid T7-gC1qR and induced with IPTG, overexpressed a ~33 kDa protein (induced) as compared to uninduced cells. Following lysis and sonication of the bacterial cells, the overexpressed gC1qR appeared in the soluble fraction sonicate, which was further purified on a Q-Sepharose column. **(B)** Western blotting to show immunoreactivity of recombinant gC1qR: 10 μg of recombinant gC1qR protein was run on a 12% v/v gel and transferred onto a nitrocellulose membrane, which was probed with anti-gC1qR polyclonal antibody. BSA was used as a negative control protein.

Recombinant expression of human gC1qR has been previously reported in *E. coli* as fusion to Glutathione-S-transferase (28). Here, we expressed human gC1qR without any fusion partner under a strong bacteriophage T7 promoter. *E. coli* BL21 (λDE3) cells, containing the gC1qR construct, expressed a ~33 kDa protein following IPTG induction, compared to the uninduced cells (Figure 1A). The overexpressed protein appeared in the soluble fraction following cell lysis and sonication. Recombinant gC1qR was subsequently purified using Q-Sepharose. It was step-eluted at 0.45 M NaCl and appeared as a single band on SDS-PAGE under reducing conditions. The immunoreactivity of the purified recombinant gC1qR was confirmed by western blot using anti-gC1qR polyclonal antibodies that were raised against native human gC1qR (Figure 1B).

### Recombinant gC1qR Binds Differentially to the Three Globular Head Modules of Human C1q

**Figure 2:**
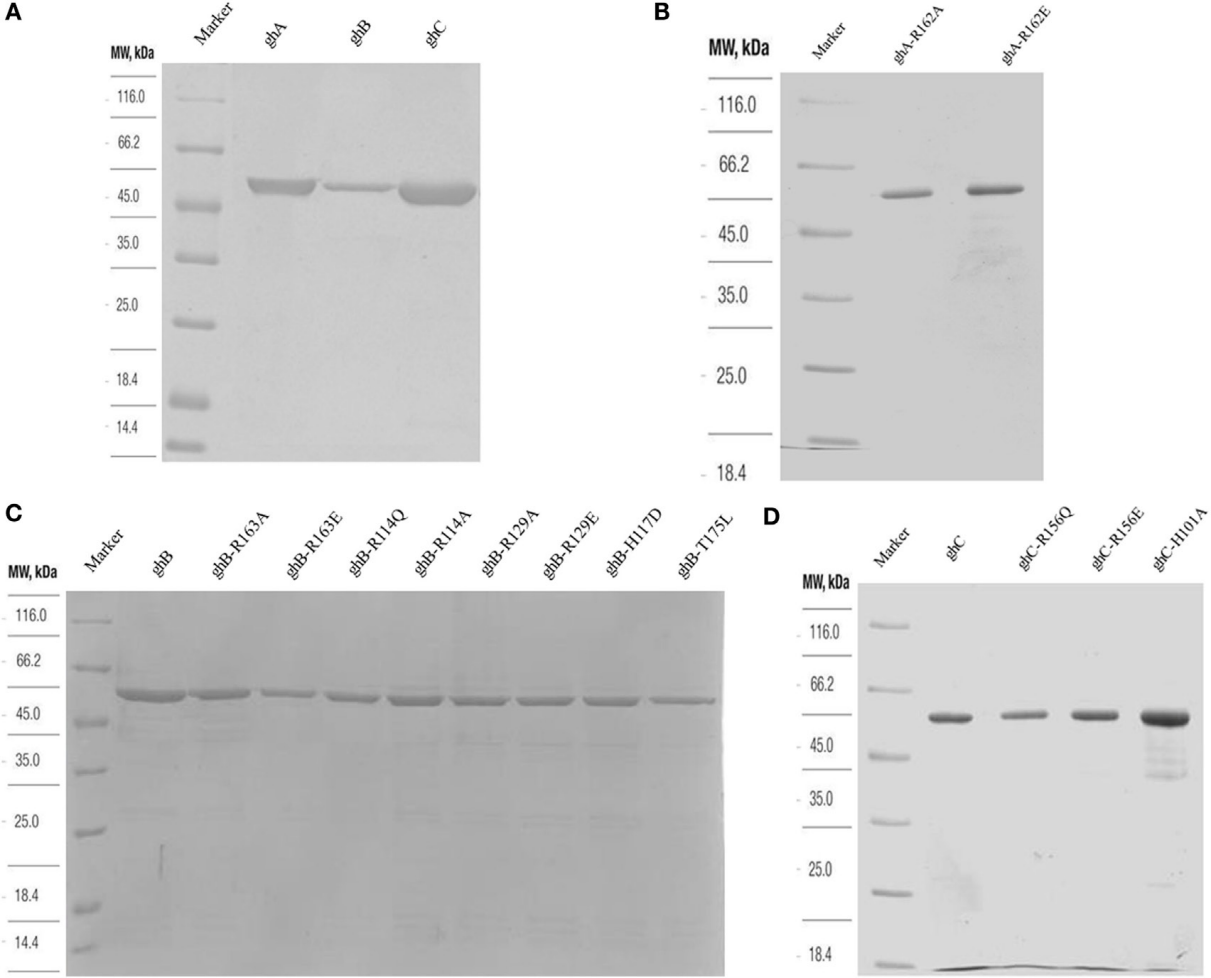
**SDS-PAGE (12% w/v) under reducing conditions of purified fusion proteins following affinity chromatography**. MBP fusion proteins containing wild type and mutant globular head modules were purified using an amylose resin column **(A)**. Purified ghA, ghB and ghC **(B)** purified substitution mutants of ghA module; **(C)** purified mutants of ghB module, R163A, R163E, R114Q, R114A, R129A, R129E, H117D, and T175L; **(D)** purified ghC, R156Q, R156E, and H101A.

**Figure 3:**
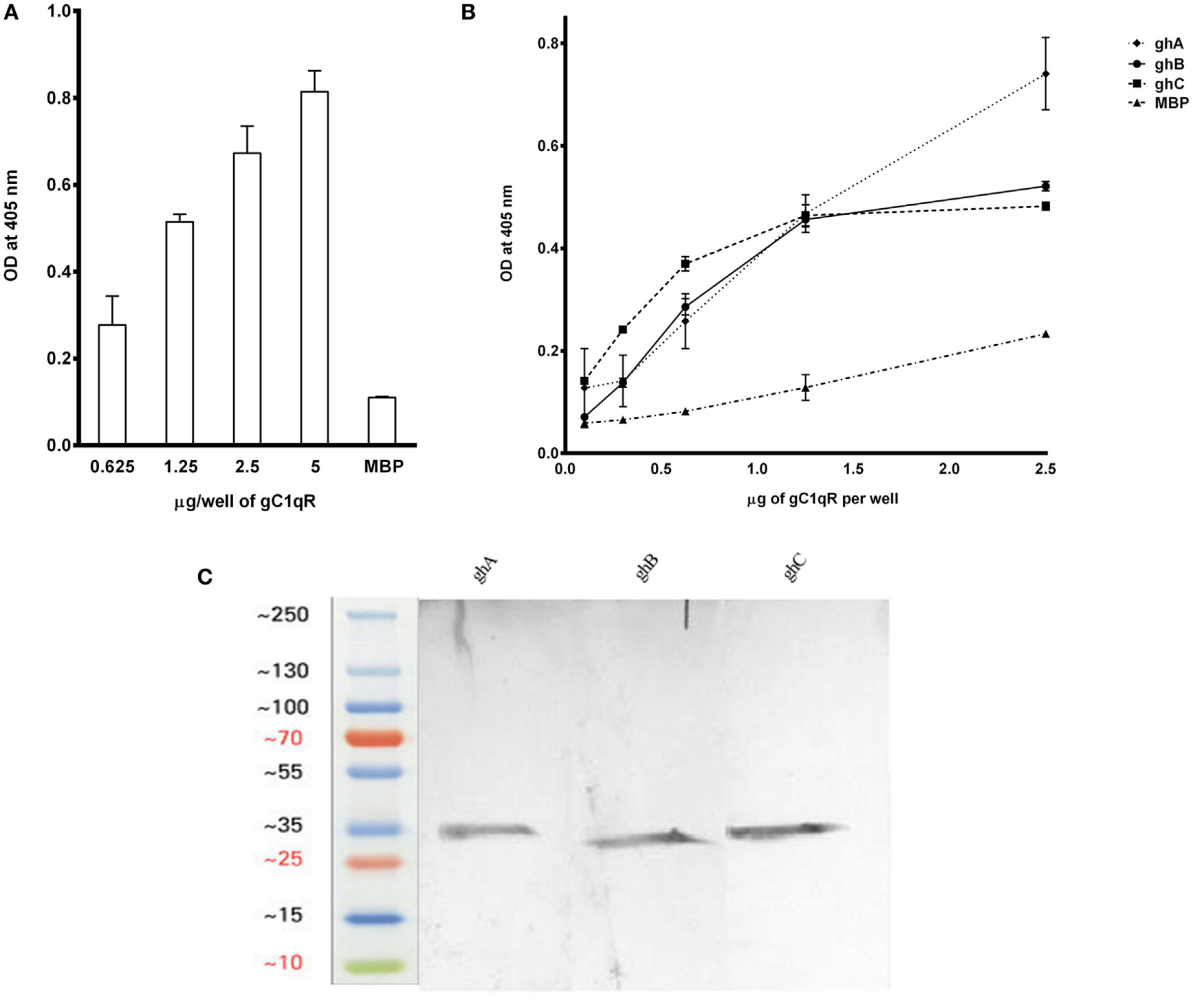
**(A)** ELISA to show binding of gC1qR to C1q: microtiter wells, coated with different concentrations of human C1q (5, 2.5, 1.25, and 0.625 μg/well), were incubated with 2.5 μg of gC1qR. Bound gC1qR was detected by anti-gC1qR polyclonal antibody followed by Protein A-HRP conjugate. MBP was used as a negative control protein. **(B)** ELISA to assess interaction of gC1qR with ghA, ghB, and ghC modules. gC1qR (1 μg/well in 100 μl) was coated on microtiter wells and then incubated with various concentrations of wild type of ghA, ghB, and ghC. MBP was used as a negative control. Following washing, bound protein was detected using anti-MBP monoclonal antibody and goat anti-mouse IgG HRP conjugate. **(C)** Far-western blot analysis to show ghA, ghB, and ghC binding to gC1qR: 15 μg of gC1qR was first run on the SDS-PAGE under reducing conditions, transferred onto a PDVF membrane and then probed with 10 μg each of ghA, ghB, and ghC. Lanes 1 through 3 show interaction of gC1qR with ghA, ghB, and ghC, respectively.

The recombinant gh modules ghA, ghB, ghC (Figure 2A) and their substitution mutants (Figures 2B–D) were expressed as MBP fusion proteins and purified on amylose resin. gC1qR bound full-length C1q in a dose-dependent manner (Figure 3A). When different concentrations of the ghA, ghB, and ghC modules were allowed to bind to a constant concentration of gC1qR, all modules bound to gC1qR independently in a dose-dependent manner (Figure 3B). ghA showed greater binding at 2.5 μg when compared with the other two modules, which is consistent with previous findings (29), which implicated ghA to be the most important gh region in the C1q–gC1qR interaction. To confirm the ELISA results, far-western blot was performed using recombinant gC1qR, ghA, ghB, and ghC proteins. Transferring gC1qR onto PDVF membrane and probing with gh modules revealed independent binding of ghA, ghB, and ghC to gC1qR (Figure 3C). Similarly, ghA, ghB, or ghC transferred onto PDVF membrane and individually probed with gC1qR confirmed the interaction (data not included) that each gh module binds specifically to gC1qR irrespective of their immobilized orientation on the membrane.

### Arg^A162^ Is Crucial for C1q–gC1qR Interaction

**Figure 4:**
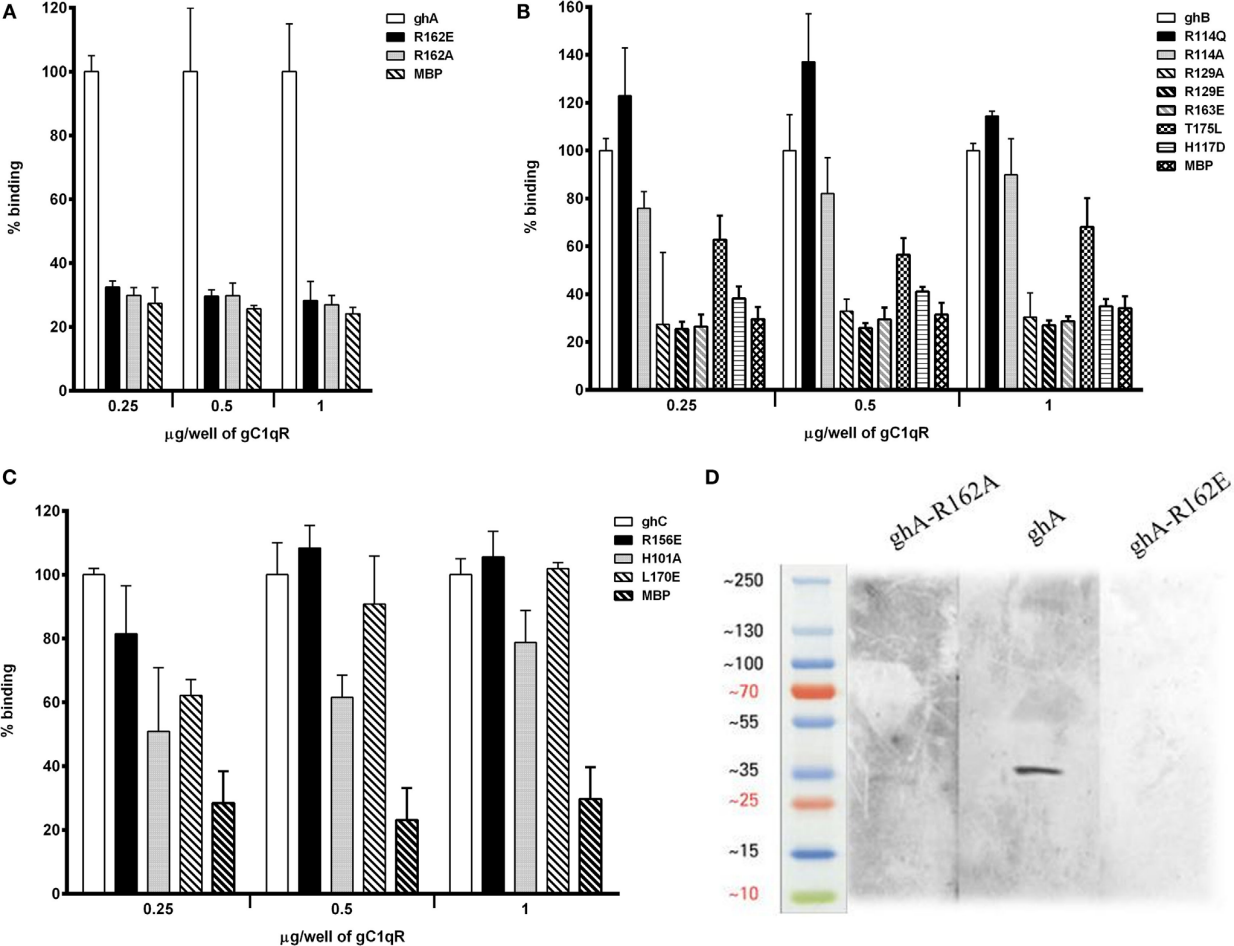
**ELISA to assess interaction between gC1qR and gh substitution mutants**. Microtiter wells were coated with different quantities (0.25, 0.5, and 1 μg/well) of gC1qR. After blocking and washing, the wells were incubated with 2.5 μg/well of **(A)** ghA, R162A and R162E; **(B)** ghB, R114A, R114Q, R163A, R163E, R129A, R129E, T175L, and H117D; and **(C)** ghC, for 90 min at 37°C and 90 min at 4°C. Bound proteins were detected with anti-MBP monoclonal antibody followed by goat anti-mouse IgG–HRP conjugate. Data are representative of three experiments. **(D)** Ligand blot to show binding of ghA mutants R162A and R162E to gC1qR: PVDF membrane strips containing gC1qR were reacted with ghA, R162A and R162E, and then probed with anti-MBP monoclonal antibody followed by goat anti-mouse IgG–HRP.

Using ELISA, we examined the ability of gC1qR to interact with recombinant ghA and its single residue substitution mutants, ghA-R162A (i.e., Arg^A162^Ala) and R162E. Different amounts of gC1qR were coated on microtiter wells and incubated with wild-type ghA, R162A and R162E. As shown in Figure 4A, the substitution of Arg^A162^ to Ala (R162A) resulted in up to 70% reduction in gC1qR binding at 1 μg/ml concentration, with respect to the background binding to MBP control protein. Substitution of Arg^A162^ with Glu (R162E) resulted in similar abrogation of binding when compared to wild-type ghA. To further confirm these observations, we carried out a far western blot (Figure 4D). Thus, 15 μg of gC1qR was transferred onto a PDVF membrane and probed with 10 μg each of ghA, R162E and R162A. Figure 4D shows a clear band for wild-type ghA. However, probing with the mutants revealed a very faint band for R162A while no band could be detected on the blot in the case of R162E.

### Arginine and Histidine Residues within the ghB Module Appear Important for Stabilizing C1q–gC1qR Interaction

The ability of ghB and its single residue substitution mutants (R114Q, R114A, R163A, R163E, T175L, R129A, R129E, and H117D) to bind microtiter-coated gC1qR was examined using ELISA. The mutant Arg^B114^ to Gln (R114Q) bound better to gC1qR than the wild-type ghB (Figure 4B), suggesting that replacing a charged (polar) residue with an uncharged residue can strengthen binding between the two proteins. Substituting Arg^B114^ with Ala, however, was not comparable with R114Q (Figure 4B). Out of all the ghB mutants, R114A, R129E, R163E, and H117D showed considerable reduction in binding. Thus, substituting arginine with glutamine had an adverse effect on the ghB–gC1qR interaction. Substituting His to Asp reduced ghB affinity for gC1qR by nearly 60%, which suggests that His^B117^ is very important for gC1qR binding. When comparing all the ghB substitution mutants, it was evident that the most significant effect was caused by the substitution of arginine to glutamine, suggesting that Arg^B163^ is crucial for gC1qR binding to C1q (Figure 4B). Similar observations were noted for the ghC mutants (Figure 4C).

### Microscopy Studies

**Figure 5:**
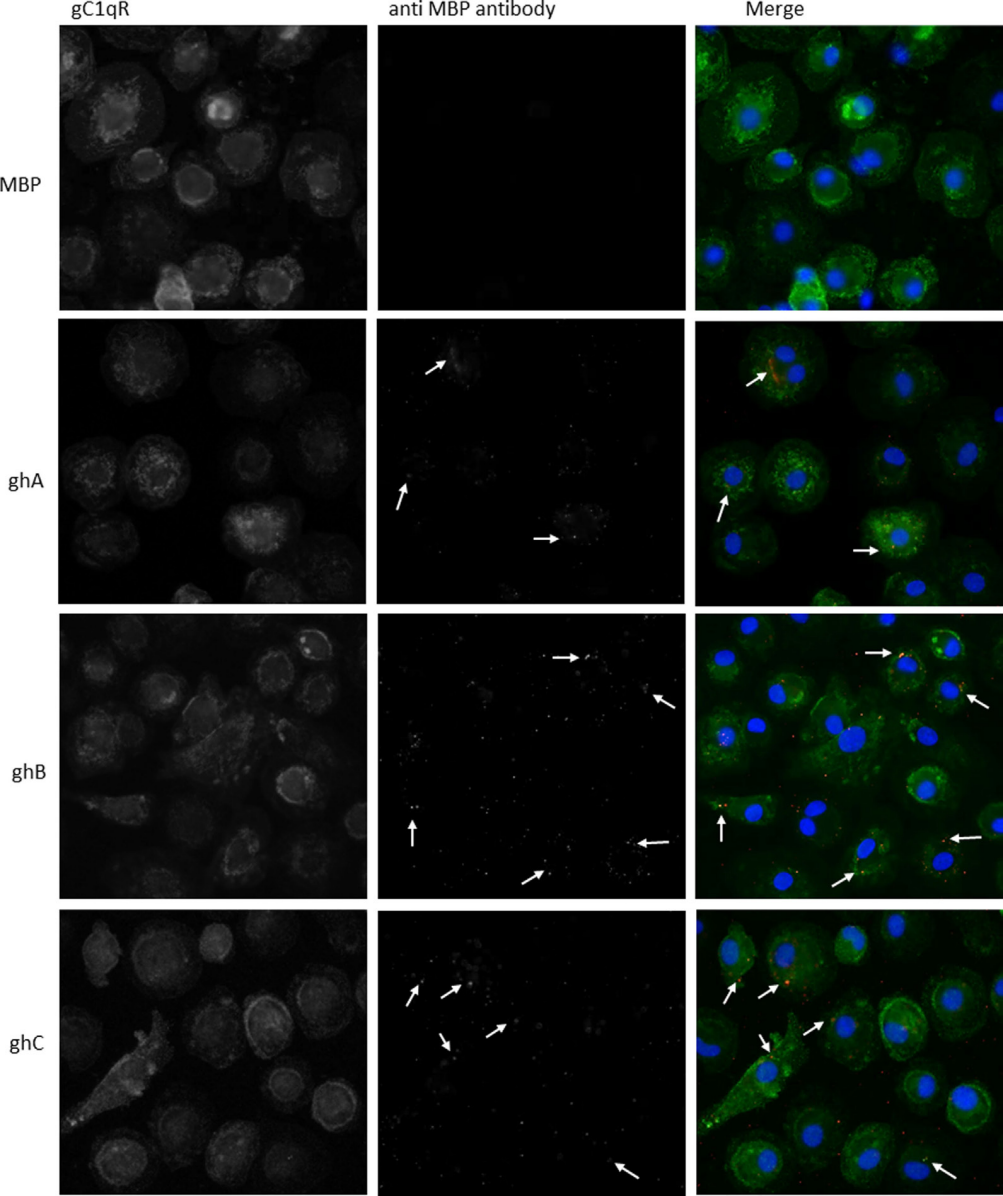
**Interaction of ghA, ghB, and ghC with monocyte/macrophages**. PMBCs (1 × 10^6^) were seeded on 13 mm coverslips and incubated in complete RPMI 1640 medium for 2 weeks at 37°C in 5% CO_2_ incubator. Cells were treated with 10 μg of each globular head module and incubated with serum-free RPMI 1640 medium for 1 h at 37°C. After washing with PBS, cells were fixed with 4% PFA, permeabilzed with Triton X-100, and probed with anti-gC1qR polyclonal antibody and anti-MBP monoclonal antibody to reveal gC1qR and bound globular head modules, respectively. Cells were washed and treated with Alexa Fluor 488 conjugated secondary goat anti-rabbit antibody and Alexa Fluor 647 conjugated secondary donkey anti-mouse antibody and the nucleus was stained with Hoechst 33342. Cells were then examined under Leica fluorescence microscope with 40× magnification. In the merged images gC1qR is green; globular heads are red; and nucleus is blue. Arrows point to bound globular heads with colocalization of globular heads and gC1qR seen in orange in the merged images. Scale bars: 10 μm.

Since we established that ghA, ghB, and ghC individually bind to gC1qR, we performed microscopic studies to determine whether the gh’s of C1q can colocalize with gC1qR on the surface of PBMCs. We first verified the interaction by identifying gC1qR on the surface using polyclonal antibodies against gC1qR (gC1qR in Figure 5). Incubation of the gh modules and probing with anti-MBP monoclonal antibodies showed the gh’s bound on the surface of PBMCS with partial colocalization to gC1qR (Figure 5, arrows in merged images).

### Transcriptional Expression of gC1qR and C1q in Adherent Monocytes

**Figure 6:**
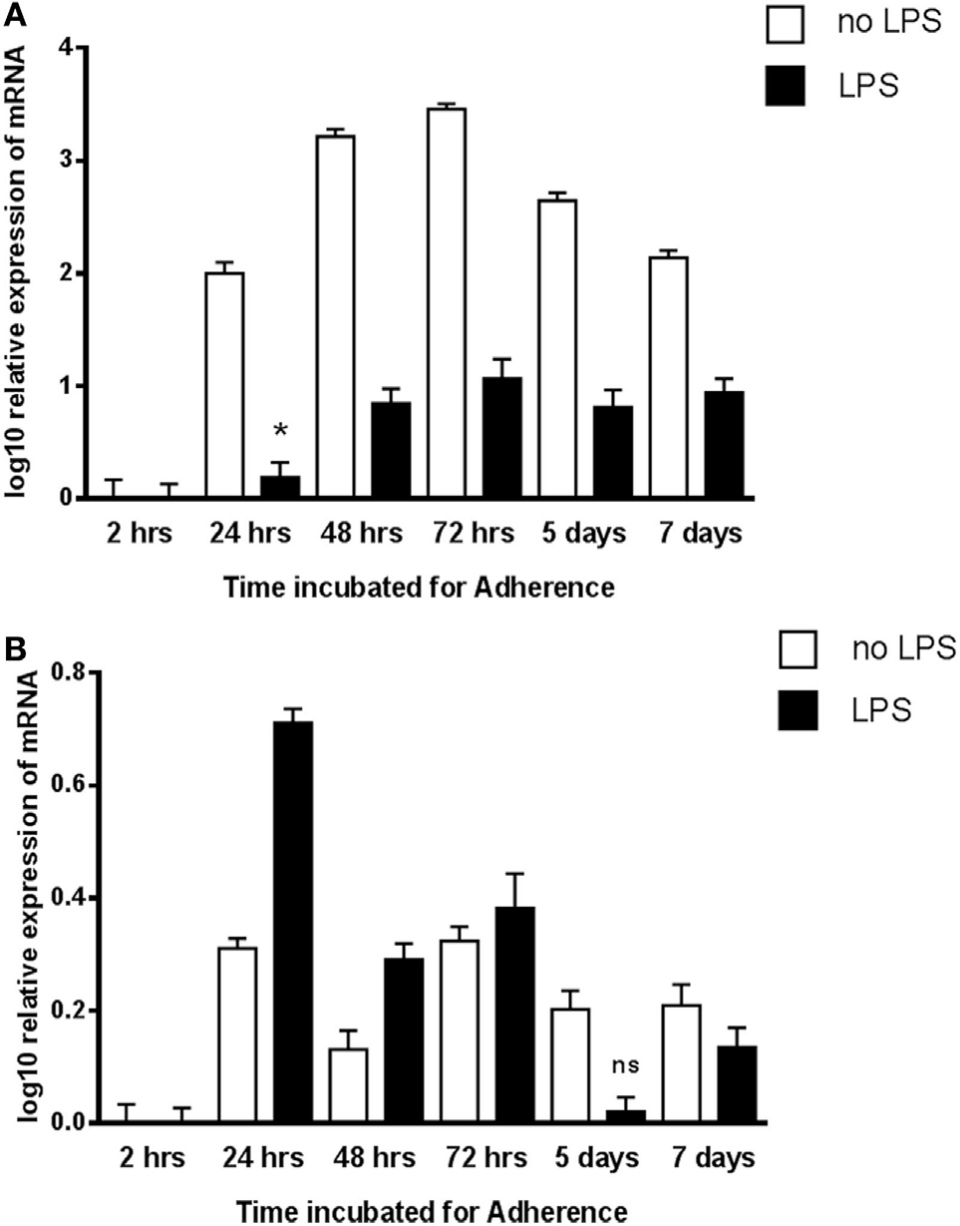
**Expression of C1q (A) and gC1qR (B) by adherent human monocytes *in vitro***. The expression of C1q and gC1qR was measured using real-time qPCR and the data were normalized *via* 18S rRNA gene expression as a control. Relative expression (RQ) was calculated by using the 2 h time point as the calibrator. The RQ value was calculated using the formula: RQ = 2^−∆∆Ct^. Assays were conducted twice in triplicates. Error bars represent ±SEM. A two-side *t*-test was performed on the data. All samples showed significant expression compared to the calibrator (*p* ≤ 0.01), except where noted: *0.01 < *p* < 0.05; ns: not significant (*p* ≥ 0.05). LPS was added to cultures at a 20 ng/μl concentration.

To examine a possible correlation between the temporal pattern of expression of C1q and gC1qR, qPCR analysis was performed in view of the fact that adherent monocytes upregulate C1q expression, a situation that mimics inflammation. In addition, the expression of C1q and gC1qR in the adherent monocytes was also assessed with and without LPS challenge (acting as a pro-inflammatory stimulus). C1q-RNA expression increased markedly in monocytes during adherence, peaking at 72 h with a log_10_ 3.5-fold difference compared to 2 h after adherence (Figure 6A). In contrast, incubation with LPS had a suppressive effect on C1q expression. There was also an increase in the gC1qR expression during monocyte adherence, with the pattern of expression appearing to be biphasic in nature with the peaks observed at 24 and 72 h (Figure 6B). In contrast to C1q, the presence of LPS elevated the expression of gC1qR, peaking at 24 h adherence, more than twice the level observed without LPS. These results are consistent with an earlier study, showing enhancement of gC1qR surface expression on endothelial cells after 24 h (30). However, LPS seems to cause enhancement of gC1qR expression rather than inhibition, unlike C1q (Figure 6A), suggesting that gC1qR on its own may have a regulatory role in LPS-mediated inflammation.

### ghA, ghB, ghC, and gC1qR Inhibit PHA-Stimulated Proliferation of PBMCs

**Figure 7:**
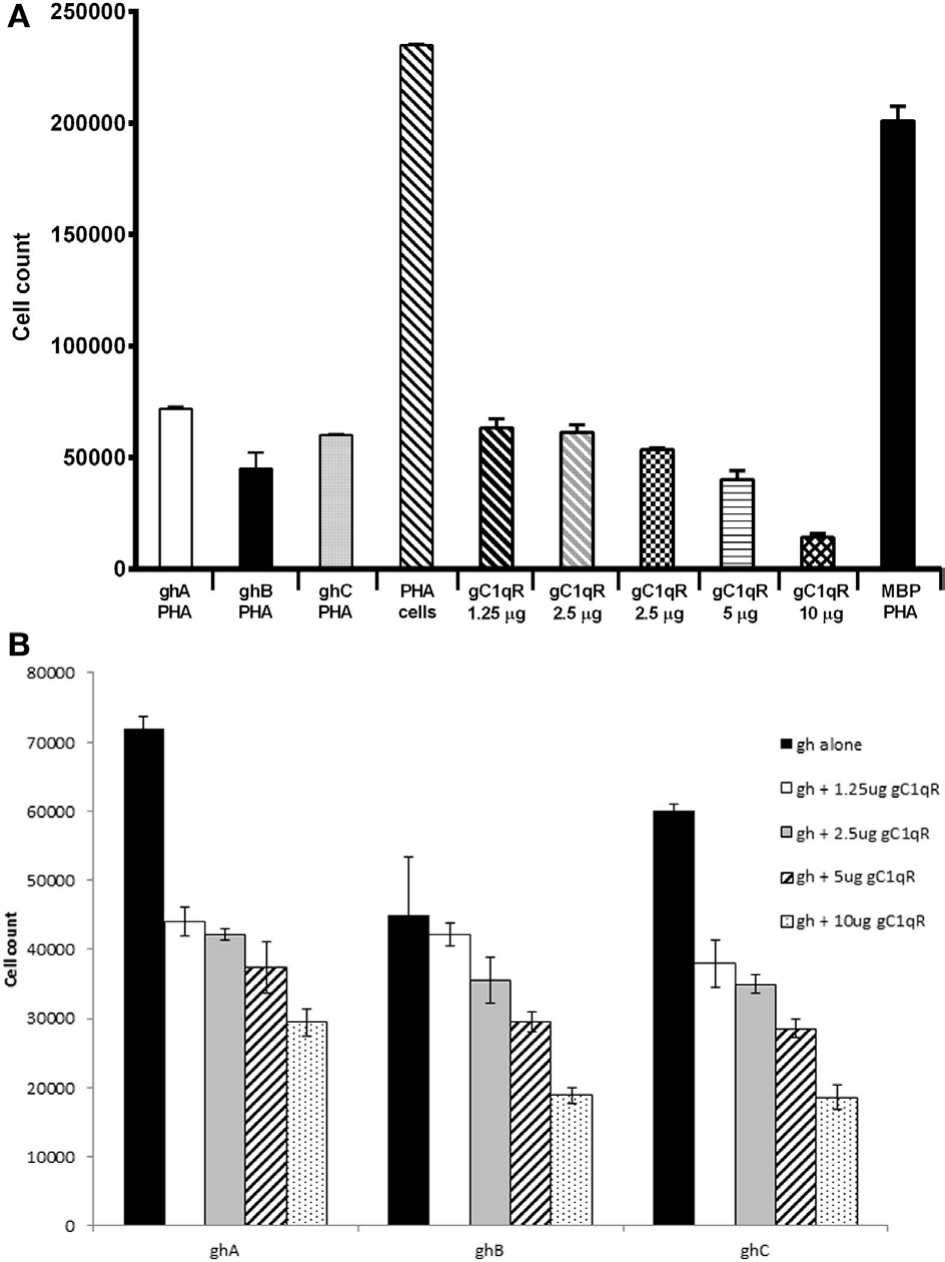
**Anti-proliferative effects of ghA, ghB, and ghC modules**. Human PBMCs from healthy individuals were stimulated with PHA (1 μg/ml) for 3 days with or without recombinant gC1qR (1.25, 2.5, 5, 10, and 20 μg/ml), ghA, ghB, or ghC (20 μg/ml) followed by a 16 h pulse in the presence of tritiated methyl thymidine ([^3^H]TdR). Each experiment was carried out in triplicates. **(A)** Each of ghA, ghB, ghC, and gC1qR inhibited PHA-stimulated proliferation of PBMCs; **(B)** ghA, ghB, and ghC coincubated with gC1qR inhibit PHA-stimulated proliferation of PBMCs.

Since C1q is known to have anti-proliferative effect on T cells (25), we examined if this inhibition is mediated through the gC1q domain. ghA, ghB, and ghC individually were able to inhibit PHA-stimulated proliferation of PBMCs (Figure 7A), as measured by thymidine uptake. The gh modules inhibited proliferation by >threefold when compared to PBMCs stimulated with PHA alone and MBP control. Next, we sought to determine whether the addition of different concentration of gC1qR to a fixed gh concentration would further inhibit proliferation. Figure 7B shows an inhibitory dose response for each gh module with the addition of gC1qR at different concentrations. The addition of 1.25 μg of gC1qR increased ghA and ghC mediated anti-proliferative effects by 40% (Figure 7B).

## Discussion

C1q, as a charge pattern recognition protein, is able to bind to a range of self and non-self-ligands through its heterotrimeric gC1q domain (5, 31). The gC1q domain is also found in a range of non-complement proteins (22, 32, 33), including collagen VIII, precerebellin, and multimerin (2, 32–37). The crystal structure analysis of the gC1q domain shows a sphere-shaped, heterotrimeric arrangement with the N- and C-terminal ends of each domain residing at the base of the trimer (5). The crystal structure has also identified an exposed Ca^2+^ ion located at the apex; this has been considered important in the binding of gC1q to its ligands.

Out of several candidate receptors for C1q, calreticulin (cC1qR) binds to the collagen region of C1q, while gC1qR interacts with the gC1q domain (2, 13). However, the nature and the context of interaction between C1q and gC1qR have not been examined. Previous studies, using ligand blot analysis with C1q run under reducing conditions, have shown that gC1qR binds predominantly to the A chain and moderately to the C chain of C1q with the A chain Arg residue at positions 162 being critical for binding (28). The availability of the recombinant forms of ghA, ghB, and ghC (18) and their substitution mutants (22) gave us with the opportunity to fully explore the gC1q–gC1qR interaction.

Previous studies have shown that gC1qR inhibits aggregated IgG-mediated complement activation by binding to the gC1q site on C1q, thereby preventing IgG from binding to the gh’s (28), suggesting that the binding sites for gC1qR and IgG on C1q may be identical or at least overlapping. It has also been shown that one of the IgG binding sites resides on Arg^162^ of the A chain (38), consistent with the mutational studies where the mutant R162E showed reduced the ability of C1q to bind to IgG by 35% (22). The importance of arginine residues in the ligand recognition of gC1q domain is consistent with the observation in this study (Figure 3B). Ghebrehiwet et al. have previously shown that the Arg^162^ in C1q A chain is significant in gC1qR binding (39). A peptide corresponding to the A chain with the Arg residue at position 162 substituted to Glu showed no binding to gC1qR. It has also been noted that residues 154–165 of the A chain are implicated in IgG binding (38), and inhibition studies have demonstrated that forming a complex between gC1qR and C1q prevented C1q binding to SRBCs, and hence, complement activation. These data are consistent with Figure 4A, which shows that substitution of the arginine residue in ghA reduces its binding to gC1qR considerably. We further examined the contributions of Arg^114^, Arg^163^, and His^117^ (of ghB) and Arg^156^ (of ghC) to the C1q–gC1qR interaction by substituting them with either neutral or negatively charged residues. Thus, the substitution of Arg to glutamine at amino acid 114 position of the B chain showed a much better binding to gC1qR than the wild-type ghB. The observation that a substitution mutant interacts better than the wild type is of great interest, offering an opportunity to neutralize the involvement of gC1qR in infection and inflammation. Experiments involving chemical modification have shown Arg^11^ of the B chain to be an important residue in IgG binding (38).

The qPCR-based mRNA expression studies appeared to suggest that both C1q and gC1qR are upregulated in adherent monocytes in a biphasic manner. It is likely that the two proteins are coexpressed under pro-inflammatory conditions and can regulate each other’s functions, or may have distinct functions by recognizing unique self and non-self-molecular targets. When LPS was used in the assay, the expression of C1q was down-regulated, but gC1qR levels were upregulated, suggesting that C1q and gC1qR may have distinct roles in LPS-mediated immune response that are independent of each other.

Since C1q is known to exert an anti-proliferative effect on T cells (25), it was of interest to examine if individual gh modules were also anti-proliferative and if the addition of gC1qR in the assay could modulate this effect. Here, we show that ghA, ghB, and ghC modules also possess anti-proliferative properties of C1q (Figure 7). gC1qR had an additive effect on the anti-proliferative effects of gh modules in a dose-dependent manner (Figure 7A). The engagement of C1q with gC1qR, expressed on CD4^+^ and CD8^+^ T cells, is known to suppress cell proliferation (25). Therefore, further attenuation of cell proliferation by gC1qR is likely to be mutually beneficial for the immune regulation. Previously, it was thought that the “full-length” gC1qR (residues 1–282) was mostly resident in the mitochondria, which is then cleaved off to release the “mature form” (residues 74–282) expressed on the cell surface (16). However, subsequent studies have shown that the membrane associated-form is, in fact, the full-length gC1qR from which the mature form consisting of residues 74–282 is cleaved off and released as a soluble form into the pericellular milieu. On endothelial cells, the soluble form has been shown to induce bradykinin 1 receptor (B1R) expression by binding to surface bound fibrinogen in an autocrine manner (40). Thus, the soluble gC1qR is capable of binding to immune cells and modulating various cellular immune responses, including cell proliferation. The anti-proliferative activity of gC1qR has also been demonstrated through the HCV core protein, which binds gC1qR on T cells and inhibits their growth, highlighting a role for gC1qR in host immune evasion (41). It is likely that the interaction of C1q with gC1qR provides a negative growth signal, which interferes with normal cell proliferation. This could prove useful in developing targeted therapies where uncontrolled proliferation of immune cells is a critical pathological issue.

The expression of gC1qR has been detected in several cellular compartments such as mitochondria (42), nucleus (43), and on the cell surface of neutrophils, mast cells, T and B lymphocytes, endothelial cells, monocytes, and platelets (44–48). However, since gC1qR is devoid of a transmembrane domain, it has been proposed to exert its signal across the membrane through a docking/signal interaction with CD44 (49). We carried out microscopy studies to investigate the colocalization of gC1qR with the gh modules on the cell surface of monocyte-derived macrophages, which revealed gC1qR’s presence on the cell surface (Figure 5). We could also observe colocalization of individual gh with gC1qR. Although C1q has been shown to bind to residues 76–93 of gC1qR, it appears that there could be additional binding sites on gC1qR (50) involving residues 144–162. This paper together with the other studies, establishing the importance of C1q–gC1qR interaction in disease models, where complement activation is a critical factor in disease progression such as atherosclerosis and Alzheimer’s disease, could be relevant for the development of novel therapeutic strategies.

## Author Contributions

LP, AP, BP, AT, AK, LK, SA, and GS carried out crucial experiments; HK, MS, and EP provided important reagents and facilities; UK and BG collaboratively designed and supervised most of the experiments, in addition to writing the manuscript.

## Conflict of Interest Statement

The authors declare that the research was conducted in the absence of any commercial or financial relationships that could be construed as a potential conflict of interest. The reviewer RS and handling Editor declared their shared affiliation, and the handling Editor states that the process nevertheless met the standards of a fair and objective review.
